# Health Care Providers’ Profiles and Evaluations of a Statewide Online Education Program for Dissemination of Clinical Evidence on HIV, Hepatitis C Virus, and Sexually Transmitted Disease: Cross-Sectional Study

**DOI:** 10.2196/10722

**Published:** 2019-03-28

**Authors:** Dongwen Wang, Meredith Abrams

**Affiliations:** 1 Arizona State University Scottsdale, AZ United States

**Keywords:** information dissemination, online systems, continuing education, HIV, hepatitis C, sexually transmitted diseases, multimedia

## Abstract

**Background:**

Timely and effective dissemination of the latest clinical evidence to health care providers is essential for translating biomedical research into routine patient care. Online platforms offer unique opportunities for dissemination of medical knowledge.

**Objective:**

In this study, we report the profiles of health care providers participating in the New York State HIV-HCV-STD Clinical Education Initiative online program and their evaluations of the online continuing professional development courses.

**Methods:**

We compiled professional and personal background information of the clinicians who completed at least one online course. We collected their self-reported program evaluation data with regard to the course content, format, knowledge increase, and impact on clinical practice.

**Results:**

We recorded a total of 4363 completions of 88 online courses by 1976 unique clinicians during a 12-month study period. The clinicians’ background was diverse in terms of demographics, education levels, professional disciplines, practice years, employment settings, caseloads, and clinical services. The evaluation of online courses was very positive (*usefulness/relevance,* 91.08%; *easy comprehension,* 89.09%; *knowledgeable trainer,* 92.00%; *appropriate format,* 84.35%; *knowledge increase,* 48.52%; *intention to use knowledge,* 85.26%; and *plan to change practice,* 21.98%). Comparison with the reference data indicated that the online program successfully reached out to the primary care communities. Both the younger generation and the senior health care providers were attracted to the online program. High-quality multimedia resources, flexibility of access, ease of use, and provision of continuing professional development credits contributed to the initial success of this online clinical education program.

**Conclusions:**

We have successfully characterized a diverse group of clinicians participating in a statewide online continuing professional development program. The evaluation has shown effective use of online resources to disseminate clinical evidence on HIV, hepatitis C virus, and sexually transmitted disease to primary care clinicians.

## Introduction

### Background

Timely and effective dissemination of the latest clinical evidence to health care providers is essential for translating biomedical research into routine patient care [1]. Dissemination and implementation science has become a priority of the national health research agenda in the United States [2] and been advocated by researchers in other countries [3-5]. With the development of information and communication technologies, online platforms offer unique opportunities for medical knowledge dissemination, owing to advantages such as wide availability, rapid outreach, flexibility in resource access and use, and cost efficiency [6-8].

Since 2008, the New York State Clinical Education Initiative (CEI) program has developed hundreds of multimedia learning modules, online continuing medical education (CME) and continuing nursing education (CNE) courses, interactive case simulation tools, and other online resources [9-10]. These resources have been disseminated to tens of thousands of health care providers from more than 170 countries [11-12]. In this study, we report the profiles of health care providers participating in the CEI online education program and their evaluations of the online CME/CNE courses. As part of a larger initiative for dissemination of clinical evidence through integrated technologies, the data presented here provide important information about the participating clinicians as well as their assessment of the content, format, effectiveness, and impact of a specific category of online resources. The results from this study will guide future research on targeting interprofessional audiences and specific approaches of online resource development for more effective knowledge dissemination.

### Dissemination of Clinical Evidence Through Information Technology

Previous research found that the traditional methods for dissemination of clinical evidence to health care providers were mostly ineffective [13]. Limitations of traditional approaches included difficulty in outreaching to geographically remote areas [7], concerns from health care providers regarding the inflexibility of access and the associated costs [8], and variations in fidelity for information delivery [14].

Early initiatives to use information technology for dissemination research generated mixed results [15,16]. With the wide use and acceptance of the internet and digital media, studies in recent years showed more effective application of online platforms for dissemination of medical knowledge to clinicians, including improvement in usefulness of information, satisfaction to format and learning environment, increase of knowledge and skills, and intention to use knowledge in practice [17-20]. Many of these dissemination initiatives had associated continuing professional development credits, which proved an important incentive to health care providers [21]. However, most of the reported findings were from pilot studies with a small sample of participating clinicians (typically in a single health profession), involving only one or a few clinical topics and a limited number of learning modules. Few studies assessed the profiles of the participating clinicians. Here we report a study of a large-scale, statewide online clinical education program for a variety of health care professionals on a comprehensive set of clinical topics related to HIV, hepatitis C virus (HCV), and other sexually transmitted diseases (STDs). Our study focused on the personal and professional profiles of the participating clinicians and their assessment of the online CEI CME/CNE courses from the perspectives of content, format, knowledge increase, and impact on clinical practice.

### Clinical Research in HIV, Viral Hepatitis C, and Other Sexually Transmitted Diseases

The HIV pandemic has been a serious threat to global public health for decades. According to the Joint United Nations Programme on HIV/AIDS, there were 1.8 million new diagnoses, 1 million deaths, and 36.7 million people living with HIV worldwide in 2016 [22]. In the United States, the Centers for Disease Control and Prevention (CDC) estimated that 1.1 million people were living with HIV and 39,782 individuals were newly diagnosed in 2015 [23]. Although we have made significant progress in treatment and prevention after decades’ fight against HIV [24-25], the scale and severity of the problem are still daunting.

In recent years, HCV also has increasingly become a major concern of public health. CDC estimated 33,900 acute HCV cases in 2015 and 3.5 million people with chronic HCV in the United States [26]. HIV and HCV co-infection is frequently seen, as are co-infections of HIV and other STDs [27-28]. The total medical costs associated with the diagnosis, treatment, and prevention of STDs were estimated to be US $16 billion per annum [29].

HIV research has advanced very rapidly since the 1990s, with many completed and ongoing clinical trials on treatment, prevention, and behavioral intervention [30]. More than 100 clinical practice guidelines have been developed over the years [31]. On average, there is a new or updated guideline every few months. Recent advances in HCV medications have made the treatment shorter, less difficult to tolerate, and more effective [32]. With the frequently updated clinical evidence, effective dissemination of the latest medical knowledge to community health care providers working on the frontline to fight HIV, HCV, and other STDs has become an essential requirement.

### New York State HIV-HCV-STD Clinical Education Initiative

The CEI program [33] is sponsored by the New York State Department of Health, with additional support from other federal, regional, and local resources. It started in 1993 as a traditional, in-person continuing medical professional education program focusing on HIV. The target audience of the CEI program is primary care clinicians such as physicians, nurses, nurse practitioners, case managers, and social workers, who are currently providing or plan to provide care to HIV patients. The program aims to increase access and quality of HIV care, expand the base of clinicians who can effectively manage HIV patients, disseminate the latest clinical guidelines, and foster partnerships between community-based care providers and HIV specialists. Over the years, the CEI in-person program has successfully trained thousands of clinicians.

To leverage the latest development in information technologies and to explore the opportunities offered by the widely used digital platforms, we initiated the CEI online program in 2008. We collaborated with domain experts across the nation and developed a large repository of online resources, including hundreds of multimedia learning modules, online CME/CNE courses (the focus of this study), and guideline-driven interactive case simulation tools [9-10,34]. We disseminated these resources through multiple channels, including a main website, a mobile website, mobile apps, online social networks, rich site summary feeds, and email newsletters [11-12,35-36]. Over a period of 6 years since its launch, the CEI website has recorded nearly 200,000 visit sessions and 1 million pageviews by audiences from over 170 countries around the world. The various CEI mobile apps have been downloaded 10,000 times. Since 2013, the program scope has expanded to include HCV and other STDs to address the emerging challenges in the field. By 2015, the program had 5000 registered clinician users. The CEI website is now consistently ranked by Google and other search engines as a top site for HIV, HCV, and STD clinical education.

## Methods

**Figure 1:**
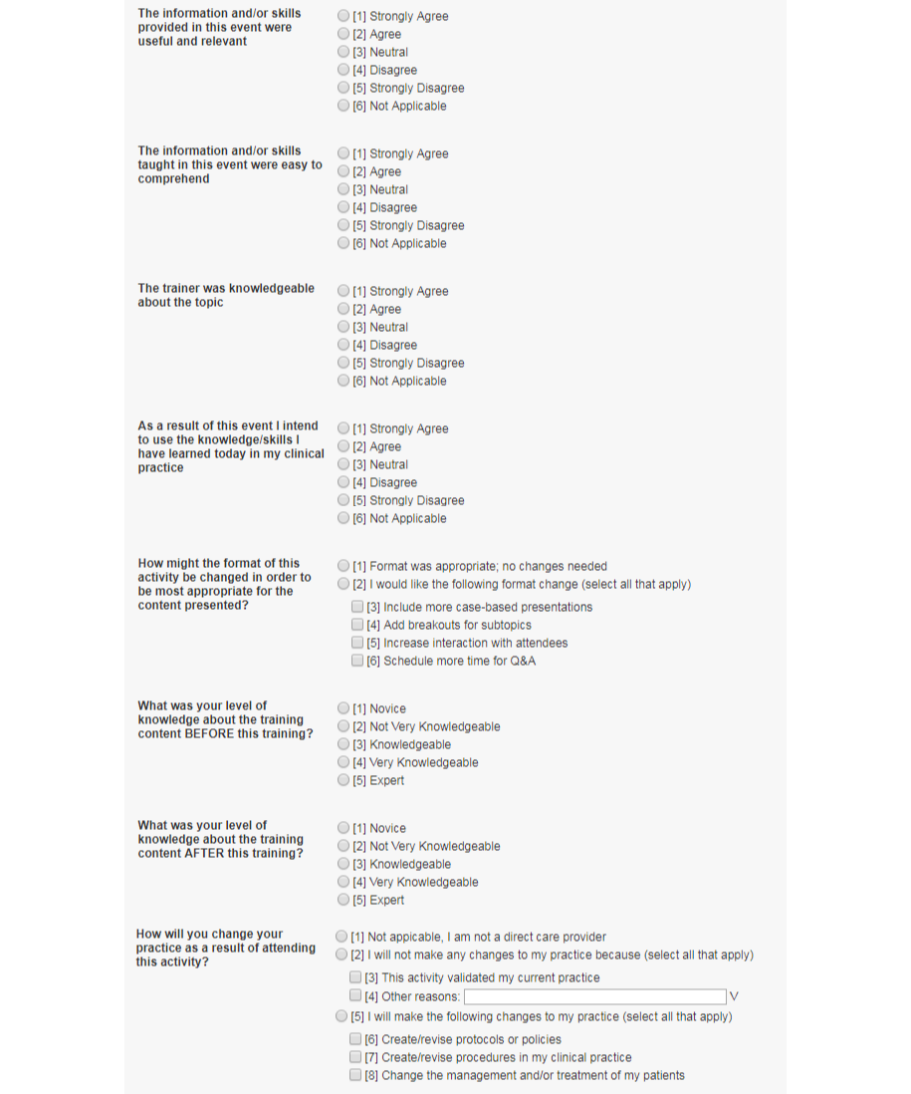
A partial screenshot of the Clinical Education Initiative Student Portal to collect course evaluation data.

**Figure 2:**
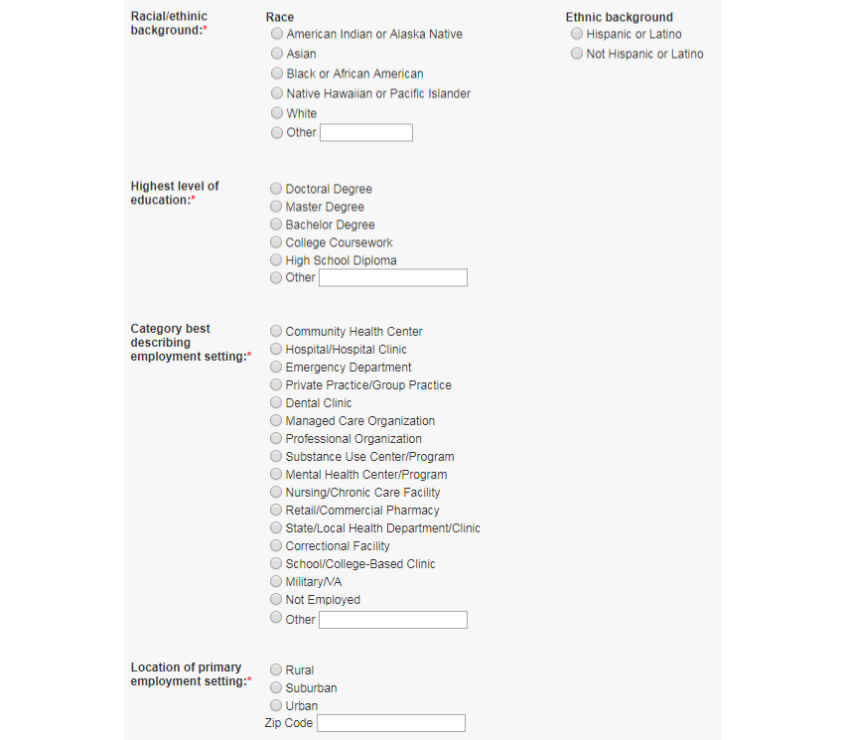
A partial screenshot of the Clinical Education Initiative Student Portal to collect student profile data.

To compile the clinicians’ profiles and their evaluations of the CEI online CME/CNE courses, we queried the CEI Student Portal and obtained data of: (1) all clinicians who successfully completed at least one online CME/CNE course from November 2014 through October 2015; and (2) their evaluations of all completed online CME/CNE courses during this period. The CEI Student Portal is a centralized system to manage clinician students, online courses, and program evaluations. A clinician user can sign up for the student portal at any time. As part of the registration, clinicians need to provide their personal (contact information, demographics, and education levels) and professional (clinical discipline, employment setting, practice years, patient caseload, and clinical services) background information. When taking an online CME/CNE course, a clinician must complete watching the multimedia materials and pass a short exam to test his/her knowledge related to the topics covered by the course. After completion of each online CME/CNE course, the clinician student must provide an evaluation with self-reported data on: (1) usefulness/relevance of the information presented in the course; (2) ease of comprehension; (3) knowledge of the trainer; (4) appropriateness of the online training format; (5) knowledge level on the course topic before and after the training; (6) intention to utilize the learned knowledge; and (7) plan to change practice after the training. Here, for items (1), (2), (3), and (6), we used a five-point Likert-scale measure (*strongly agree*, *agree*, *neutral*, *disagree*, and *strongly disagree*), and the responses were further grouped into positive (*strongly agree* and *agree*) and nonpositive (*neutral*, *disagree*, and *strongly disagree*) responses for data analyses. For item (5), we used five discrete levels to indicate a clinician’s knowledge on a specific training topic (*novice*, *not very knowledgeable*, *knowledgeable*, *very knowledgeable*, and *expert*) before and after the training, and then calculated the difference (*at least one level increase* vs *no increase*) for data analyses. In addition to these structured questions for course evaluation, clinicians can provide free-text comments on their experiences of the CEI online training program. Once the evaluation is completed, the CME/CNE credits associated with an online course are immediately awarded. At any time, the clinician student can review and print the CME/CNE certificate. Partial screenshots of the CEI Student Portal to collect course evaluation and student profile data are shown in Figures 1 and 2.

For data analyses, we categorized the online CME/CNE courses based on a custom-developed domain ontology of HIV-HCV-STD clinical topics [9]. We then profiled all clinicians’ backgrounds and computed their evaluations of the online CME/CNE courses with the seven measures discussed above. As the last step, we reviewed the themes from the free-text comments to capture additional feedback for the CEI online training program. This study was approved by the University of Rochester Research Subjects Review Board and Arizona State University Institutional Review Board.

## Results

We recorded a total of 4363 completions of 88 online CME/CNE courses by 1976 unique clinicians during the study period. Among these clinicians, 1333 (67.46%) were female, 1103 (55.82%) were white, 492 (24.90%) were African American, 128 (6.48%) were Asian, and 436 (22.06%) were Hispanic. In terms of the clinicians’ education level, 308 (15.59%) held a doctoral degree and 486 (24.60%) held a master’s degree. The details of the clinicians’ demographics and education levels are shown in Table 1.

The top categories of the recorded primary professional discipline were physician (n=257, 13.01%), nurse (n=229, 11.59%), case/care manager (n=219, 11.08%), nurse practitioner (n=148, 7.49%), and social worker (n=148, 7.49%). Regarding the practice years in the primary professional discipline, 124 (6.28%) indicated more than 30 years of experience, 224 (11.34%) between 21 and 30 years, 402 (20.34%) between 11 and 20 years, and 1182 (59.82%) 10 years or less. For clinicians’ employment setting, the top categories recorded were community health center (n=379, 19.18%), hospital/hospital clinic (n=350, 17.71%), state/local health department/clinic (n=190, 9.62%), private/group practice (n=169, 8.55%), managed care organization (127, 6.43%), and professional organization (n=108, 5.47%). Among these, 1257 (63.61%) were in urban settings, 439 (22.22%) were in the suburbs, and 280 (14.17%) were in rural areas. Details of the clinicians’ primary professional disciplines, years in practice, and employment settings are provided in Table 2.

For the caseload, 171 (8.65%) clinicians reported seeing more than 100 HIV patients per month, 82 (4.15%) reported seeing more than 100 HCV patients per month, and 108 (5.47%) reported seeing more than 100 STD patients per month. The caseload category of 1-10 patients per month recorded the largest number of clinicians (n=515, 26.06% for HIV; n=466, 23.58% for HCV; and n=454, 22.98% for STD). The top clinical services provided for HIV patients were risk reduction intervention (n=617, 31.22%), case management (n=456, 23.08%), and HIV screening/testing (n=447, 22.62%). The top services for HCV patients were risk reduction intervention (n=538, 27.23%), HCV screening/testing (n=381, 19.28%), and HCV treatment (n=179, 9.06%). The most popular services for STD patients were risk reduction intervention (n=623, 31.53%), STD screening/testing (n=359, 18.17%), and STD treatment (n=289, 14.63%). The details of the clinicians’ caseloads and clinical services are provided in Table 3.

Regarding the number of online CME/CNE courses taken by the health care providers, 1454 (73.58%) clinicians completed only one course, 182 (9.21%) completed two, and the remaining 340 (17.21%) clinicians completed three or more. Excluding a CEI staff who worked as a system tester, the largest recorded number of online CME/CNE courses completed by a single clinician was 47, and the average number of courses taken was 2.17.

As a measurement of course popularity, we recorded a list of courses with the most numbers of completions. The top three on the list were: (1) *HIV/AIDS Confidentiality Law Overview*, with 1379 completions; (2) *Street Drugs & HIV*, with 195 completions; and (3) *STD-HIV Inter-Relationship*, with 158 completions. A complete list of the top 20 most popular online CME/CNE courses is shown in Table 4.

In terms of course evaluation, the overall responses from the clinicians were very positive. With the 4363 completed courses, 3974 responses (91.08%) indicated that the information presented was useful and relevant, 3887 (89.09%) indicated that the course was easy to comprehend, 4014 (92.00%) indicated that the trainer was knowledgeable, and 3680 (84.35%) indicated that the online training format was appropriate. With regard to the impact of the online training, 2117 of the 4363 responses (48.52%) indicated at least one level increase in knowledge, 3720 (85.26%) indicated intention to use the learned knowledge, and 959 (21.98%) indicated a plan to change clinical practice. Excluding those who did not provide direct patient services at the time, 39.43% (959/2432) of those who responded planned to change their clinical practice after the training. A summary of the course evaluation data is shown in Table 5.

**Table 1:** Participating clinicians’ demographics and education levels.

Characteristics		n (%)
**Gender**		
	Male	632 (31.98)
	Female	1333 (67.46)
	Transgender - male to female	4 (0.20)
	Transgender - female to male	7 (0.35)
**Racial background**		
	American Indian or Alaska Native	20 (1.01)
	Asian	128 (6.48)
	Black or African American	492 (24.90)
	Native Hawaiian or Pacific Islander	7 (0.35)
	White	1103 (55.82)
	Other	226 (11.44)
**Ethnic background**		
	Hispanic/Latino	436 (22.06)
	Not Hispanic/Latino	1540 (77.94)
**Highest level of education**		
	Doctoral degree	308 (15.59)
	Master degree	486 (24.60)
	Bachelor degree	575 (29.10)
	College coursework	315 (15.94)
	High school diploma	153 (7.74)
	Other	139 (7.03)

**Table 2:** Participating clinicians’ primary professional disciplines, years in practice, and employment settings.

Characteristics		n (%)
**Primary professional discipline/occupation**		
	Case/care manager	219 (11.08)
	Counselor	95 (40.81)
	Dental hygienist	2 (0.10)
	Dentist	9 (0.46)
	Health educator	80 (4.05)
	Health profession student/trainee	56 (2.83)
	Health program administrator/coordinator	97 (4.91)
	Lab manager/technician	5 (0.25)
	Medical/dental assistant	22 (1.11)
	Nurse	229 (11.59)
	Nurse practitioner	148 (7.49)
	Nutritionist/dietician	11 (0.56)
	Pharmacist	25 (1.27)
	Pharmacy technician	8 (0.40)
	Physician	257 (13.01)
	Physician assistant	27 (1.37)
	Psychiatrist	2 (0.10)
	Psychologist	8 (0.40)
	Public health professional	74 (3.74)
	Social worker	148 (7.49)
	Therapist/interventionist	8 (0.40)
	Other	446 (22.57)
**Years in primary profession/occupation**		
	>30 years	124 (6.28)
	21-30 years	224 (11.34)
	11-20 years	402 (20.34)
	0-10 years	1182 (59.82)
	Unknown	44 (2.23)
**Employment setting**		
	Community health center	379 (19.18)
	Hospital/hospital clinic	350 (17.71)
	Emergency department	15 (0.76)
	Private practice/group practice	169 (8.55)
	Dental clinic	10 (0.51)
	Managed care organization	127 (6.43)
	Professional organization	108 (5.47)
	Mental health center/program	75 (3.80)
	Nursing/chronic care facility	44 (2.23)
	Substance use center/program	16 (0.81)
	Retail/commercial pharmacy	12 (0.61)
	State/local health department/clinic	190 (9.62)
	Correctional facility	19 (0.96)
	School/college-based clinic	25 (1.27)
	Military/veterans affairs	7 (0.35)
	Not employed	54 (2.73)
	Other	376 (19.03)
**Location of primary employment setting**		
	Rural	280 (14.17)
	Suburban	439 (22.22)
	Urban	1257 (63.61)

A total of 288 comments from the clinicians were collected during the study period. Review and analysis of these comments excluded 156 invalid or meaningless responses. For the remaining 132 comments, we further categorized them into five themes: information/content, format, trainer, technical issues, and general positive feedback, with 4 belonging to two separate themes. Among these, 39 indicated that the clinicians learned a lot from the presentation and found it very informative. For example, a comment under this theme was “the information is useful for updating agency policy,” indicating that the clinician planned to utilize the learned information in their facility. The 53 general positive feedbacks such as “I like it” and “excellent program” only expressed clinicians’ overall positive experiences but without details. Additional positive comments were recorded on knowledgeable trainer (n=3) and appropriate online format (n=1). We recorded 25 comments about technical issues encountered by the clinicians, for example, “video was cut off at 40 minutes’ mid-sentence.” Other negative comments included challenging materials or other information issues (n=4), problem with the trainer (n=5), and request for improvement of the format (n=6).

**Table 3:** Participating clinicians’ caseloads and clinical services.

Characteristics		n (%)
**Number of patients per month - HIV/AIDS**		
	0	778 (39.37)
	1-10	515 (26.06)
	11-20	127 (6.43)
	21-40	146 (7.39)
	41-60	117 (5.92)
	61-100	122 (6.17)
	>100	171 (8.65)
**Number of patients per month - HCV^a^**		
	0	1071 (54.20)
	1-10	466 (23.58)
	11-20	127 (6.43)
	21-40	109 (5.52)
	41-60	70 (3.54)
	61-100	51 (2.58)
	>100	82 (4.15)
**Number of patients per month - STD^b^**		
	0	1019 (51.57)
	1-10	454 (22.98)
	11-20	146 (7.39)
	21-40	104 (5.26)
	41-60	85 (4.30)
	61-100	60 (3.04)
	>100	108 (5.47)
**Clinical services - HIV/AIDS**		
	Adherence counseling	340 (17.21)
	Case management	456 (23.08)
	Medication management	300 (15.18)
	Postexposure prophylaxis	191 (9.67)
	Pre-exposure prophylaxis	234 (11.84)
	HIV screening/testing	447 (22.62)
	Mental health management	237 (11.99)
	Partner services	180 (9.11)
	Peer education	282 (14.27)
	Resistance testing	163 (8.25)
	Risk reduction intervention	617 (31.22)
	Screening for opportunistic infections	141 (7.14)
	Other	162 (8.20)
	None	824 (41.70)
**Clinical services - HCV**		
	HCV screening/testing	381 (19.28)
	HCV treatment	179 (9.06)
	Risk reduction intervention	538 (27.23)
	Other	94 (4.76)
	None	1175 (59.46)
**Clinical services - STD**		
	Partner services	190 (9.62)
	Physical assessment	217 (10.98)
	Risk reduction intervention	623 (31.53)
	Screening/testing	359 (18.17)
	Treatment	289 (14.63)
	Vaccination	200 (10.12)
	Other	96 (4.86)
	None	1119 (56.63)

^a^HCV: hepatitis C virus.

^b^STD: sexually transmitted disease.

**Table 4:** The top 20 most popular online continuing medical education/continuing nursing education courses.

Course	Completions, n (%)
HIV/AIDS Confidentiality Law Overview	1379 (31.61)
Street Drugs & HIV	195 (4.47)
STD^a^-HIV Inter-Relationship	158 (3.62)
The Clinical Diagnosis and Treatment of Gonorrhea, Chlamydia, and Genital Herpes	114 (2.61)
Sexual Assault Evaluation: What Health Professionals Need to Know	101 (2.31)
HIV Pre-Exposure Prophylaxis in the Real World	92 (2.11)
HIV and Aging	90 (2.06)
The Clinical Diagnosis and Treatment of Syphilis	84 (1.93)
Diagnosis and Treatment of Acute HIV: A Stitch in Time?	77 (1.76)
Smoking Cessation in the HIV Patient	70 (1.60)
HIV Prevention and Care in Transgender People	65 (1.49)
Vaginitis	61 (1.40)
Advances in the Treatment and Prevention of HIV Infection: CROI^b^ 2015, Focus on ART^c^	60 (1.38)
HIV Sexual Networks: Transmission Dynamics, and Drug Resistance	59 (1.35)
The Patient Protection and Affordable Care Act: What It Means to Patients	56 (1.28)
Drug-drug Interactions in HIV and HCV^d^ in an Aging Population	55 (1.26)
Management of HIV/HCV Co-infection in 2014: Cure for All?	55 (1.26)
The Good, the Bad and the Ugly of Inflammation in HIV Infection	53 (1.21)
Clinical Management of Alcohol Use & Abuse in HIV-Infected Patients	50 (1.15)
What Is It with HIV-2?	48 (1.10)

^a^STD: sexually transmitted disease.

^b^CROI: Conference on Retroviruses and Opportunistic Infections.

^c^ART: antiretroviral therapy.

^d^HCV: hepatitis C virus.

**Table 5:** Clinicians’ evaluation of the online continuing medical education/continuing nursing education courses.

Measures and responses					n (%)
**Information useful and relevant (n=4363)**					
	**Positive**				3974 (91.08)
		Strongly agree		2358 (54.05)	
		Agree		1616 (37.04)	
	**Nonpositive**				389 (8.92)
		Neutral		339 (7.77)	
		Disagree		19 (0.44)	
		Strongly disagree		15 (0.34)	
		Not applicable		16 (0.37)	
**Easy to comprehend (n=4363)**					
	**Positive**				3887 (89.09)
		Strongly agree		2190 (50.19)	
		Agree		1697 (38.90)	
	**Nonpositive**				476 (10.91)
		Neutral		420 (9.63)	
		Disagree		34 (0.78)	
		Strongly disagree		13 (0.30)	
		Not applicable		9 (0.21)	
**Knowledgeable trainer (n=4363)**					
	**Positive**				4014 (92.00)
		Strongly agree		2495 (57.19)	
		Agree		1519 (34.82)	
	**Nonpositive**				349 (8.00)
		Neutral		304 (6.97)	
		Disagree		16 (0.37)	
		Strongly disagree		11 (0.25)	
		Not applicable		18 (0.41)	
**Format appropriate (n=4363)**					
	**Positive**				3680 (84.35)
		Format appropriate, no change needed		3680 (84.35)	
	**Nonpositive**				683 (15.65)
		**I would like the following format changes**		683 (15.65)	
			Include more case-based presentations (n=683)	382 (55.93)	
			Add breakouts for subtopics (n=683)	96 (14.06)	
			Increase interactions with attendees (n=683)	112 (16.40)	
			Schedule more time for Q&A (n=683)	61 (8.93)	
**Intend to use knowledge (n=4363)**					
	**Positive**				3720 (85.26)
		Strongly agree		2155 (49.39)	
		Agree		1565 (35.87)	
	**Nonpositive**				643 (14.74)
		Neutral		440 (10.08)	
		Disagree		15 (0.34)	
		Strongly disagree		16 (0.37)	
		Not applicable		172 (3.94)	
**Change of knowledge level after the training (n=4363)**					
	**Positive**				2117 (48.52)
		4		28 (0.64)	
		3		35 (0.80)	
		2		422 (9.67)	
		1		1632 (37.41)	
	**Nonpositive**				2246 (51.48)
		0		2160 (49.51)	
		–1		68 (1.56)	
		–2		13 (0.30)	
		–3		3 (0.07)	
		–4		2 (0.05)	
**Will change practice (n=4363)**					
	**Positive**				959 (21.98)
		**I will make the following changes to my practice**		959 (21.98)	
			Create/revise protocols or policies (n=959)	448 (46.72)	
			Create/revise procedures in my practice (n=959)	232 (24.19)	
			Change the management of my patients (n=959)	187 (19.50)	
	**Nonpositive**				3404 (78.02)
		**I will not make changes to practice because**		1473 (33.76)	
			This training validated my current practice (n=1473)	415 (28.17)	
			Other reasons (n=1473)	1289 (87.51)	
		Not applicable - no current patient service		1931 (44.26)	

## Discussion

In this study, for the first time, we reported a detailed profile of health care professionals participating in a statewide online HIV-HCV-STD knowledge dissemination program. Although there were limited reference data, we had a few interesting findings. First, compared to the data of general health care workforce in New York State [37], the proportion of the CEI program participants working in nonhospital community settings was much higher (82% vs 58%). Comparison to the data of active registered nurse in New York State [38] showed similar results (82% vs 29%). These data demonstrated that the CEI online program successfully reached out to the primary care communities, as defined by its missions. Second, 14% of the CEI program participants were from rural areas, similar to the active registered nurse data in New York State (14%) [38]. Further research is required to assess the detailed geographical distribution of the participants and the potential changes over time. Third, the demographics of the CEI program participants indicated a slightly more racially/ethnically diversified workforce (56% white, 25% black, 6% Asian, and 22% Hispanic) as compared to the data of active registered nurses in New York State (61% white, 18% black, 12% Asian, and 6% Hispanic) [38]. This perhaps reflected the higher incidence and prevalence of HIV, HCV, and STD infections in minority groups and therefore the need for more racial/ethnic diversity in health care provider teams. Future research is required to explore race and ethnicity as co-variates for clinician’s course evaluation.

To relieve the potential concern on privacy, we did not collect CEI participant’s date of birth. Instead, we used years of practice as an alternative measure, which we believed could roughly estimate a health care provider’s age in most cases. The data showed that a majority (60%) of the CEI audience had 10 years or less of experience in practice. This result was consistent with previous finding that an online program tended to attract younger clinicians [14]. In addition, 18% of clinicians in the program had more than 20 years of working experience, indicating that this group of health care providers was actively engaged in the CEI online program.

Similar to a previous pilot study [39], we recorded data of clinicians from a variety of professional disciplines participating in the CEI online program. As increasingly recognized and advocated by the research community [40], the interprofessional approach for knowledge dissemination defined another contribution of our study. Related to that, we identified a comprehensive set of clinical services provided by the professionals participating in the CEI online program. Future research is required to assess potential interactions among professional disciplines, clinical services, training topics, and course evaluations.

With regard to caseload, our data indicated a wide range of distribution, from 0 to 100+ HIV, HCV, and STD patients per month. The largest category of the participating clinicians had 1-10 patients per month, a typical scenario in the primary care setting. It is interesting to note that 4%-9% of the clinicians had 100+ patients per month, indicating that the CEI online program also attracted HIV, HCV, and STD specialists, as suggested by anecdotal evidence. In addition, 39%-54% of the clinicians had no patients at the time—they likely were those: (1) in the HIV, HCV, and STD clinical care and public health teams but with no direct interactions with patients; or (2) new practitioners preparing to engage in HIV, HCV, and STD patient care.

According to the Accreditation Council for Continuing Medical Education 2015 Annual Report, there were close to 37,000 enduring internet material activities that provided more than 74,000 hours of instruction to 4.8 million physicians and over 6.8 million other learners [41]. Nevertheless, just posting materials online for reading is not enough. Studies showed that multimedia materials and interactivity would increase the satisfaction of health care professionals for their participations in online learning [42]. Other important factors used by clinicians in the selection of an online course included the quality of content, flexibility of access, ease of use, and convenience of obtaining continuing professional development credits [42]. We believe that effectively addressing these issues has led to the initial success of the CEI online program.

The data from a recent national survey called for a central repository for listing educational opportunities and tracking continuing education credits [43]. Nonetheless, another study found that online materials from open public sources such as YouTube had overall low quality [44]. A large repository of centrally managed, high-quality online courses with a student portal to track the progress of learning and CME/CNE credits is likely an important contributing factor for the success of the CEI online program in engaging a core group of clinicians. Our data showed that this core group of clinicians kept coming back to the CEI online program to take new courses.

This study has a few limitations. First, we focused only on a specific category of online resource, multimedia CME/CNE course, while many other types of resources such as case simulation, InfoButton, and clinical decision support could also be leveraged for dissemination of clinical evidence [20,43,45]. As advocated by others, multi-faceted interventions will likely be more effective for knowledge dissemination [20]. The CEI online program has already developed a few other types of resources and communication channels [9-11]. Future research is required to assess the effectiveness to use multiple categories of online resources for knowledge dissemination. Second, this research was based on a cross-sectional descriptive study design without comparative groups (except for the measure of clinicians’ knowledge level, which involved a before-after comparison). Future research is required to compare the effectiveness of the interventions delivered through the online resources with specific types of controls in order to demonstrate that the benefits of online courses outweigh the disadvantages [42-43]. Third, we relied on self-reported data [46] to measure clinicians’ satisfaction on content, format, knowledge change, and potential improvement in clinical practice. Given that CEI is a statewide program, this is perhaps the best we could do. Future research should include direct measures on health care processes and patient outcomes [47]. Finally, since the CEI online program was newly developed at the time, there were still technical issues (as shown in the clinicians’ comments) during the study period. The CEI program staff were actively working to address those issues found by the clinician students. Ideally, a system should be frozen during evaluation [48]. However, considering a large program such as CEI that serves thousands of clinicians, it is unrealistic to freeze the system and ignore the identified issues for a long study period. We therefore decided to evaluate the system along the normal process of system maintenance, which reflected the real status of its performance.

In conclusion, we have successfully characterized the profiles of clinicians participating in the New York State CEI online CME/CNE program for dissemination of the latest HIV, HCV, and STD clinical evidence. We have identified a diverse group of health care providers in terms of the demographics, education levels, professional disciplines, practice years, employment settings, caseloads, and clinical services. The participating clinicians’ evaluation of the CEI online CME/CNE courses was very positive with regard to the content, format, and knowledge increase. A significant portion of the participating clinicians planned to adopt the learned knowledge and skills in their practice. These initial set of evaluation data have demonstrated the effectiveness of using online resources for dissemination of HIV, HCV, and STD clinical evidence to primary care clinicians. Future research is required: (1) to assess the effectiveness of knowledge dissemination with multiple types of online resources and using direct measures on health care processes and patient outcomes; (2) to examine the comparative effectiveness with control groups; and (3) to analyze the co-variates through development of a predictive model for effective online training.

## Abbreviations

AHRQ: Agency for Healthcare Research and Quality
CDC: Center for Disease Control and Prevention
CEI: Clinical Education Initiative
CME: continuing medical education
CNE: continuing nursing education
HCV: hepatitis C virus
STD: sexually transmitted disease
